# Veratrum Viride Again

**Published:** 1854-05

**Authors:** Wm C. Butler

**Affiliations:** East Avon


### ART. III.—*Veratrum Viride again.* By WM. C. BUTLER, East Avon.

Once upon a time an ancient philosopher, looking down from the towering summit of true science upon his enthusiastical and fanatical disciples, was led to exclaim "Save me from my friends"—knowing full well that all the vain sophistry, ingenious theory, or dire anathemas of his opponents would be swept, by the effulgent rays of truth, like mist before the morning sun. But from his professional friends, a far different source of danger was to be apprehended. In their over zeal they would draw deductions that his premises would not allow, and jump at conclusions which could not be sustained. Hence the exclamation. Look at all the really great discoverers, either in the sciences or arts, from the first dawn of creation up to the present time, and see how few could not exclaim with him, "Save me from my friends."

Your humble writer was led into the above train of thought by once listening to an aged and venerable disciple of Esculapius, whose head was whitened with the frosts of many winters, or the withering *blasts of experience,* holding the following language. He said, deep thought, and patient research, and close observation had convinced him that he had at last found a remedy which would act as a tonic, sedative, alterative, stimulant, emmenagogue, &c., &c., depending altogether upon the dose — in short, in one hand he held Pandora's box open, and ruthlessly let fly its contents to the four winds of heaven, that he might, like the cruel sportsman, bring its game prostrate at his feet with his universal panacea. And what think you, kind reader, was this magic remedy? Calomel — nothing more or less than calomel! Now I would ask, in all candor, if you were to give the sub-muriate of mercury the power of language, would it not exclaim in tones too plain to be misconstrued, "save me from this my friend?"

Can we expect that any real addition to medical science will be more fortunate in the nineteenth century than in any previous one? Far from it. Place in our therapeutical armamentaria any new and really useful remedy, and it at once assumes the position of a ripe cherry upon the tree. For this there are two reasons: 1st, no discrimination is made in cases; 2d, it is from this fact expected to do, in every case, what it has done in one. In other words, *with some there are no contra-indications.* There is no truth upon the page of medical literature more prominent than the above, and as our friend, veratrum viride, is just now passing the fiery ordeal, let us look for a moment at its true merits, in hopes the exclamation may not be applied to us.

To every reflecting mind who has had the misfortune to be located in a malarious district, where the too common notion of bilious and calomel are synonymous terms, there has long been felt the necessity of a remedy which would act as a stimulant to the glandular system; or, as Dr. Norwood more properly, perhaps, calls it, a deobstruent. Nor is it here alone so sensibly felt, as a residence in a densely populated city for a few months past has convinced me. I shall, therefore, in the present communication, call the attention of the reader to one of its many therapeutical indications, leaving the others for subsequent ones. And here let me advise him to go and quaff deeply at that deep and inexhaustible fountain of common sense and common sense principles, the venerable John Hunter. He has laid down this principle, which, with many others, has not been gainsayed, viz: that if you in any way destroy the nervous influence to an organ, you destroy, in the same ratio, the function of that organ. Now it matters little, as I conceive, how this is done: whether by malarious poison entering the circulation and depressing the whole nervous system, or by local injury affecting the special nervous influence to a certain organ, or by a local drain upon the nervous system gradually undermining the whole, and thereby bringing the glandular system, as a whole, under the general law of Hunter. It is in constitutions affected by rational causes, and producing rational results, that I propose to show the indications and contra-indications of veratrum viride. In other words, to show that when the cause is removed, be it what it may, that we may then have an effect of the cause still existing which plainly calls not for the peculiar and specific changes in the blood and glandular system which mercury produces — but which requires a simple deobstruent and tonic to the depressed nervous system in order to restore the patient to a physiological condition. Should this be done, of what incalculable value is it to the future welfare and happiness of the patient. Let me here say, that to the use of mercury I have no objection; but against its abuse, do most solemnly protest. That very seifsame John Hunter has given us another indisputable principle, to wit: that when we have frequent irritation of an organ it becomes, so to speak, the weaker organ. Hence, all things being equal, it pays the penalty of every transgression. The time, I trust, is not far distant when irritable glands, made doubly irritable by the abuse of mercury in some of its forms, shall no longer be the opprobrium of the profession as a whole; but its use, made doubly beautiful by rescuing suffering humanity from organic change, so important to their welfare, shall place it where it deservedly belongs, as one of the most valuable remedies in the materia medica.

In looking over my note-book, I see that the cases to which I shall allude in this paper are divided into four classes, depending upon the cause; and from these shall select but one from each class, unless a different principle is involved, as it is tedious to follow through case after case having strong analogies, as is too often done.

Class I. Case, Sept. 1, 1852. Mrs. G., æt. 36, mother of five living children; has had three miscarriages previous to nine years ago; since which time her health, which was previously good, has been gradually declining; first complained of lassitude and weariness upon over exercise, with pain in the back and hips, extending down the thighs; leucorrhœa constant, with painful menstruation; the function of digestion became impaired and appetite unusually capricious; lost the roseate tint of health gradually from month to month, and for the last seven years has been unable to attend to her ordinary duties; says she cannot recollect the time when she has felt *rested;* perhaps it will be well to state here that the farm of her husband joins that of her father, so that all the local causes of disease remain nearly the same. Some five years since, her friends noticed that the extreme pallor of countenance was being substituted by a dingy yellow, which has been gradually increasing ever since, at least up to the present date. Her present appearance is like one laboring under general anas arca, with a peculiar yellow dingy countenance; a peculiar listlessness; pulse 140 per minute; bellows sound of anæmia over the heart; skin dry; tongue pale and flabby; mucous membrane the same; no abnormal sounds about the lungs; liver very much enlarged, extending some two inches below the ribs; the function of the kidneys sometimes torpid, at others abundant; bowels constantly laboring under a clay colored diarrhœa; spleen also enlarged; uterus presented a congested and indurated state of the os and cervix, with abrasion and ulceration of its mucous membrane. (Dr. Lee to the contrary, notwithstanding.) So far as the patient could judge, the present state of disease has been gradually coming on since her abortions, save in extreme cold weather, when she usually felt some alleviation. Perhaps it would be well to state here, that she had been under different forms of medical treatment, including all the schisms and isms of the day, until within the last two years, when she had abandoned them all, and has been treated for as many different forms of disease as she has had different practitioners. That protean form of disease covered by the broad mantle of bilious and nervous, had been fired at without any save a temporary good, and permanently bad, effect.

*Remarks.*—In looking over this case, it will be seen that the first sensible impression was manifested in the digestive organs in the various functions of the stomach. 2d. The functions of the liver and kidneys were next deranged, and so on through the various organs of the system. From what cause? Evidently from the drain upon the nervous system, caused by the disease of the os and cervix. For we had a woman previous to these abortions in good health, with a good constitution. 3d. The system bore up under the drain upon it for two years, to a greater or less extent, and just in proportion to the local drain were the constitutional effects. After this time, she became too weak to attend to the ordinary duties of her household, and gradually sunk into the pathological condition above stated. To follow the case a little more closely: it will be seen that the first deviation from a physological condition was in the uterus; and the first organ that sympathized with this in function, was the stomach. Now, Dr. George Budd, in his admirable treatise on disease of the liver, points out these very causes as being the ones that may produce the pathological change which he calls *softening*, as being the change necessary to produce death. He says, p. 275: "I have brought together, from different sources, the cases related in this chapter, for the sake of showing that the secretion of bile may be suppressed, and the secretory substance of the liver more or less disorganized, in various circumstances, and without the occurrence of any process that we are warranted in designating *inflammation*. It would seem that this suspension of the secreting process and disorganization of the liver may result from powerful and depressing emotions, but that it is far more frequently by some poison introduced from without, or engendered in the body by *faulty digestion* or *assimilation*. It appears, too, that various poisons — as the poison of serpents — the poison of some forms of fever, and many others — (I suppose he means by this any that will produce the effect,) may alike stop the secretion of the liver, and lead to the same kind of disorganization of its structure, while their other effects on the system are far different. It is probable too, that in some cases, as in those last related, the disorganization is produced slowly and gradually, *and so without shock*, while in the more terrible forms of disease, of which instances were before given, the disorganizing process is rapid," &c. &c. He then goes on to say, that it is only fatal cases which he has given, in order to show the pathological changes; but that all the stages from the slightest deviations given, must be met with. His remarks on treatment cannot be too seriously considered; and I quote from his high authority, with pleasure, as showing that his sagacious mind looks not through a false medium.

After looking at the nitro-muriatic acid which has long been celebrated in India for its efficacy in functional derangements of the liver, he proceeds to say: "Of medicines that render the secretion of the liver more active, and thus increase the flow of bile, or, as they have been termed, *Cholagogues*, the most energetic is mercury." In the occasional bilious disorders of persons who have no organic disease of the liver, a dose of calomel, or blue pills, followed by a brisk saline purgative, produces more speedy relief than anything else — and is more likely, therefore, to prevent inflammation or ulceration of the gall-ducts, which seems generally to result from the irritation of unhealthy bile.

Occasionally and under these circumstances, and especially in persons of full habit, mercury may be given with great advantage. *But its frequent use in any case may lead to much mischief. When the liver has been accustomed to the stimulus of mercury, no other medicine will sufficiently excite its action.* The person is thus led to the habitual use of this medicine, and, after a time, the constitution is seriously injured by it. In the class of cases we have just been considering, where, from organic disease, the liver is inadequate to its office, and nutrition has suffered much in consequence, mercury, although even here it may relieve for the moment, almost invariably does harm. It increases the activity of the liver at first, but seems to leave it weaker than before; and if frequently resorted to, the nutrition of the patient, impaired by the original disease, *is still further impaired by the drug.* In all such cases, we should be content with milder medicines, which promote the secretion of bile without having any prominent deleterious effect upon the system."

Though not directly in point, I cannot forego the further pleasure of quoting the equally high authority of our distinguished countryman, Prof. Alonzo Clark, who, as a profound scholar and pathologist, is second to none of our transatlantic brethren, and of whom every American physician may well be proud. He said (in a recent conversation with him upon the abuse of mercury,) that in looking over more than three thousand prescriptions made by himself at the Bellevue Hospital, he found that mercury, in some form, entered into the combination of but TWO

*Treatment.*—This consisted in the application of the potassa cum calce to the os and cervix uteri, followed by nit. arg. until the local disease was removed. Internally a saturated solution of the veratrum, commencing with six drops once in four hours, increasing it to ten or twelve, as the stomach would bear, without nauseating it. This, with the fortieth of a grain strychnia three times a day, constituted the treatment until June 1, 1853, when she was discharged cured, and remains well up to date.

It perhaps may be urged, and justly, too, that the medicine had nothing to do with the cure—that the cause being removed, the effect must of necessity have followed. This suggested itself to me, and in from case second to six, which were counterparts of case first, though not as far advanced, I gave them local treatment first, and let them pass on from two to three months before commencing with the medicine. For the first four or six weeks, there seemed to be a gradual improvement; but from that time on, they remained stationary until they commenced with the remedy, when the change seemed to be all one could ask. In this class there are some forty cases: Eight of them were treated separately; the rest in combination.

CLASS II.—*Case.* Mr. A. æt. 36, farmer. (In this case it will be perceived that there was no uterine irritation. No, not any!) Temperate habits; has been subject to fever during the earlier periods of his life; (bilious or calomel, I care not which you call it, for if he did not commence with both, he had them before he got through;) for the last ten years has enjoyed good health; constitution strong and vigorous; in July 1847, in slipping from a stack of wheat some sixteen feet in height, alighted on a (fork's staff,) which entered at the left of the anus, taking the urethra in its course, and lodged in the right illiac fossa. Found him, about an hour afterward, in state of collapse, without the loss of more than four ounces of blood. In consultation with my distinguished friend, Dr. Edson, of Scottville, it was agreed to give him all the stimulants and tonics the stomach would bear, and see what reaction would develop. This was feebly manifested about the tenth day, and from that time on, he gradually convalesced. Some three years after, a portion of the pantaloons was discharged *via* the wound, and about one year after, some small portions of the internal table of the illeum. As the object of this case is not in a surgical but medical point of view, I have been thus brief, simply to show that the constitution felt its effect primarily through the medium of the sacral plexus of nerves. Some three years after, we find him presenting a similar train of symptoms to case first, so far as his general appearance, and the condition of the abdominal organs, (minus a uterus,) are concerned. In his case, all the various forms of alteratives were used with little or no benefit, so far as restoring him to health was concerned. Some time in September, 1852, commenced with the veratrum and strychnine. Countenance soon began to improve; liver, spleen, &c., slowly regained their healthy dimensions, and for the first time since the accident, is feeling like himself, May, 1853. In this class I have but three cases: two from injury to the spine, and the above.

Class III. In the third class of cases which I shall present in this paper, is the impression made upon the nervous system through the medium of poison introduced by the blood — commonly called miasm. From this class, the cases in which are numerous, I shall select only one, as it demonstrates the principle of the whole; and this I choose to let the patient tell in his own language, though not strictly scientific. The letter is dated Charleston, S. C., Nov. 28, 1853, and reads thus:

"Dear Doctor: I embrace this opportunity to let you know how your red drops (ver. vir.) operated on me. When I first came to you in June last, I had the Southern fever rooted and grounded in my whole system. This fever works similar to the yellow jaunders of the North. In addition to this, I had the fever and ague the worst way. After trying other medicines for a long while without any good effect, (calomel in abundance,) I came to you in a most miserable state of health. After taking the red drops for about two weeks, I began to feel stronger in my limbs. You then gave me some white drops (strychnia) for the ague and fever; (strange antidote,) and from this time on the chills began to get lighter to the second and third, which was my last. I continued your drops until the last of October, when I arrived here and commenced work at my trade the week following, and have been hard at it ever since, and am happy to say that I never felt better for business, or enjoyed my food and labor better in my life."

Signed, J. M. THOMAS.

This short epistle tells a volume connected with the appearance of the patient. Pale, sallow, haggard and careworn, his every expression bespoke a mind prostrated by an incubus that it could but contemplate, without the power to grapple with it. Despair was written on every expression, and hope, that bright harbinger of earthly joys, was to him a stranger. In short, he was laboring under the effect of a legitimate cause, producing a legitimate result, added to or aided by legalized treatment for *ague and fever*. His thoracic organs healthy; abdominal in nearly the same condition as case first. Here, again, the cause was removed by change of climate, and the remainder is for the Profession to judge as they see fit. The case of Thomas requires more than this passing notice, especially so far as the effect of the medicine is concerned. Free emesis followed the use of four drops, and, to use his own expression, "and that, too, without sickness." When taken in quantities so small that it produced no emesis, his appetite was unusually good, aud digestion perfect. Again, he regained flesh rapidly, and in proportion as his sallowness disappeared. From his history I learned that the invasion of disease was gradual, and referred more particularly to general malaise, bad digestion and assimilation at first—these gradually gaining strength until the present condition was attained. Now it strikes me that nothing could be more natural than this very effect. Located in a malarious district, where he was constantly inhaling the poison, as soon as sufficient was imbibed to have the system cognizant of the fact, its legitimate symptoms soon followed. And just such effects follow like causes everywhere. "No mystery is here." Thus far the same effect has followed the use of the medicine, in the several cases in this class—but as the cause which produced the disease, in these cases, in this vicinity, is still in operation, a relapse is rationally to be expected.

Be this as it may: time only will demonstrate whether the system will lose its susceptibilities to the impressions of the medicine.

Class IV. The fourth and last class of cases are unfortunately few in number in the country, and the true rationale is beautifully given by Bence Jones, Golding Bird, George Johnson, Liebig, and others. These truly great medical philosophers have demonstrated conclusively that in proportion as the mind is actively employed are the phosphates of the system expended. Join to this the fact that this very class of thinkers are persons of sedentary habits, and you have two fruitful sources of disease; and generally the third is a twin sister, viz., bad air. It will be conceded that it requires no very great stretch of the imagination or reasoning faculties to see imperfect digestion, mal-assimilation, dormant function of the liver, enlarged spleen, &c., follow as a legitimate effect of this train of causes; and I am informed by eminent members of our profession in the city, that it is just the train of effects that they do get. I had intended to have the history of a case which I had the pleasure of seeing with my friend Dr. Sayre from his note-book; but as his favor in answer to mine is not yet received, must beg his pardon for any imperfections from my brief notes.

*Case.*— C. T. æt. about 26 of nervo-bilious temperament; has been confined closely to the store for the last few years, where the mind is continually upon the stretch; warm air to breathe; had complained of much languor, lassitude, and weariness for the last few months; indigestion; torpid bowels, &c., &c. From a jaunt into the country, over-done, and the system manifested it by frequent chills, followed by little or no reaction. Had taken tonics, which seemed to break them up for the time being, but would relapse as soon as the patient was from under their influence. When I first saw him, the patient was laboring under the symptoms of uraemic poisoning, for which the Dr. was giving him the colchicum and acetate of potash. As soon as this was relieved, I proposed giving him the viride, and from that time on there was a satisfactory convalescence.

The above cases, though few in number, are not wholly without instruction, and the principle which they demonstrate is still more important: That there is, and ever has been, a condition of the system which has, from time immemorial, been termed bilious — and which, since the introduction of mercury, has been considered that very condition which this drug was intended to alleviate, no one will pretend to gainsay.

That the causes leading to this very effect have been just as little taken into consideration, no one will pretend to deny.

Is it not plain, then, that in those cases, and those only, in which we have inflammation of the substance or any portion of the natural organs, that mercury is indicated as possessing those qualities necessary to prevent organic change? And if we are to believe such men as Dr. Budd, (and he gives us in this no new principle,) must we not, of necessity, acknowledge that, in those cases where mercury is contra-indicated, we can expect to do nothing but harm with it? if not primarily, surely secondarily? Again: does our knowledge of veratrum viride, or any other remedy, permit us to say that it will produce those very changes so essential to prevent organic change as mercury in the inflammatory process? Surely not. What, then, it may be asked, have we gained? If nothing more, I hope the fact, that if we can do our patient no good with calomel, we will certainly do him no harm. Dr. Willard Parker, who is truly very high authority either in medicine or surgery, made the following judicious remark. He said: "I fear practitioners of medicine, as a whole, are too much like a school-boy, who is continually looking for a rule to solve his problem; when, if they would throw this all on one side, and take up the human system as the watchmaker does his watch, and see how different causes had produced this or that result — or, in other words, let the simple facts in the case demonstrate the rule, there would be less errors in diagnosis, and less blunders in practice."

If the above cases do not prove that veratrum viride has done any good, they do prove, conclusively, that in them mercury has done no harm; and, as one of our very wise critics has consigned it to the shades of the catacombs, he can make any disposition of the above facts that pleases him best. I would simply suggest that my own impressions are, that it will prove too much like one of the professors in the Buf. Med Col., viz: the lower you put him down, the higher he is sure to rise. Be this as it may, Dr. Norwood will please accept my thanks for the introduction of a remedy that thus far has not disappointed me. One great fault with many, says Dr. Parker, is that they expect medicine to do too much. Instead of directing the man of sedentary habits and high living to saw his own wood, make his own garden, or hold his own plough, and live on a plain diet — just the indications in the case — they make a drug-shop of his stomach, until mother Nature, tired of his transgressions of the physical laws, and doubly tired of the unnatural stimulus of excessive medication, yields itself into the hands of its common enemy. In looking over my note-book carefully, I am forced to the conclusion that if the ver. vir. has any influence in the cases of the several classes, it does so, in a great measure, by its action upon the digestion and assimilation. How much of this is due to strychnia, an extensive series of experiments can alone demonstrate. It is, I believe, acknowledged by almost every one, that the latter medicine is one of the best of tonics to an exhausted nervous system, where there is no inflammatory action to contra-indicate its use. It is equally true that, as in every case the cause, where it could be ascertained, was first removed, if possible — which is ever the first indication in medicine — that other remedies, such as taraxacum, hyd. of pot., &c., &c., might have done what the hellebore is supposed to have accomplished.

Again: This may be mere theory with regard to its modus operandi; but I shall consider this more at length in a subsequent paper. It is sufficient to say for the present, that if it is at all indicated, mercury is not, and vice versa. That many of its reputed properties are entirely misconceived, I have not the least doubt. And that it would have a salutary influence in many cases where it is not used, I am equally as certain.

